# The lift snorkel technique for type Ia endoleak after fenestrated endovascular aneurysm repair of a juxtarenal abdominal aortic aneurysm

**DOI:** 10.1186/s40792-021-01115-9

**Published:** 2021-01-31

**Authors:** Eisaku Ito, Takao Ohki, Naoki Toya, Hikaru Nakagawa, Ryou Nishide, Kohei Okazaki, Tadashi Akiba

**Affiliations:** 1 Department of Vascular Surgery, The Jikei University Kashiwa Hospital, Kashiwa city, Chiba prefecture Japan; 2 Division of Vascular Surgery, Department of Surgery, The Jikei University School of Medicine, 3-19-18, Nishi-shimbashi, Minato City, Tokyo, 105-8471 Japan; 3 Department of Surgery, The Jikei University Kashiwa Hospital, Kashiwa city, Chiba prefecture Japan

**Keywords:** Lift technique, Snorkel technique, Juxtarenal abdominal aortic aneurysm

## Abstract

**Background:**

The snorkel technique for a juxtarenal abdominal aortic aneurysm (JAAA) is an important treatment option for high-risk patients. We report the lift snorkel technique through the trans-femoral access for a type Ia endoleak after fenestrated endovascular aneurysm repair (FEVAR) in a case of difficult trans-brachial access.

**Case presentation:**

A 76-year-old woman who had JAAA presented with a type Ia endoleak and sac expansion after FEVAR. We planned for proximal additional stentgraft with the bilateral renal artery snorkel technique. However, during the secondary intervention, it was difficult to cannulate to the left renal artery through the trans-brachial access due to interference of the supra-renal stent. Stentgraft was eventually delivered into the left renal artery via the trans-femoral access with a 5 Fr sheath. A plain angioplasty balloon was inserted coaxially through the sheath. The balloon was inflated in the proximal end of the stentgraft and then pushed up to replace the proximal end from down to up. The additional aortic cuff was deployed parallel to the snorkel stentgraft. One year after the additional treatment, computed tomography (CT) revealed aneurysm sac shrinkage.

**Conclusion:**

The lift snorkel technique is a unique method converting the retrograde approach to antegrade renal artery stenting and would be an effective option for difficult trans-brachial cases for a type Ia endoleak after FEVAR of a JAAA.

## Background

The snorkel technique for a juxtarenal abdominal aortic aneurysm (JAAA) is an important treatment option in high-risk patients [1, 2]. However, trans-brachial stent delivery is difficult in some cases, such as those involving occlusion of the subclavian or axillary artery, the type III aortic arch, post-arch replacement, or obtuse renal artery angle [3–5]. Here, we report the lift snorkel technique through the trans-femoral access for a type Ia endoleak after fenestrated endovascular aneurysm repair (FEVAR).

## Case presentation

**Fig. 1:**
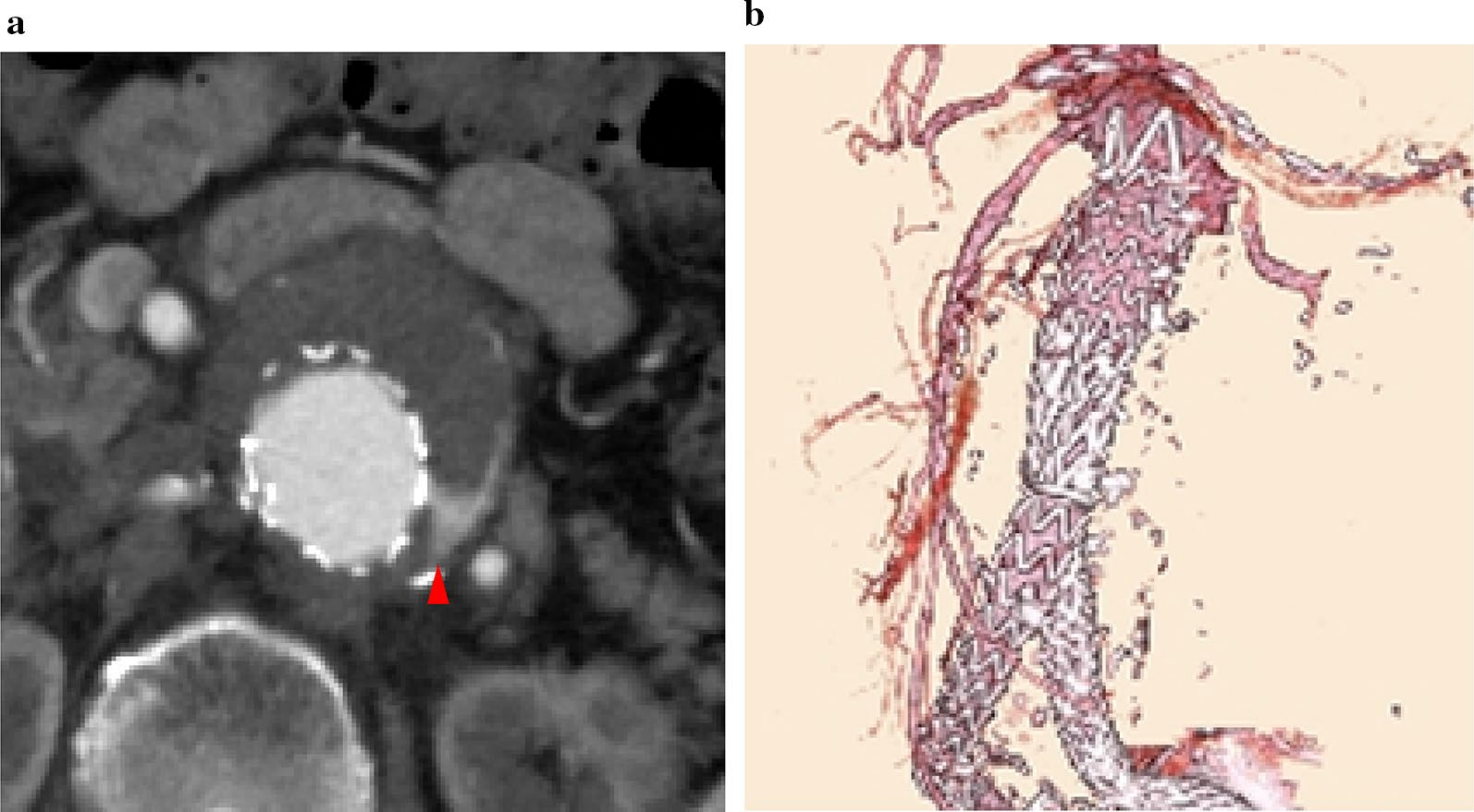
**a** The axial image of the postoperative computed tomography revealed a type Ia endoleak (arrowhead). **b** The 3D image of the postoperative computed tomography revealed a migration of the stentgraft

The patient was a 76-year-old woman who had JAAA with 28 mm proximal neck diameter and 3 mm proximal neck length of the right side, concomitant with colorectal cancer and chronic renal failure. We scheduled FEVAR, wherein the surgeon modified the left renal artery fenestration using a 32-mm-diameter Endurant II (Medtronic Inc., Dublin, Ireland) for extending the right side of the proximal neck before open surgery for colorectal cancer. Postoperative computed tomography (CT) revealed a type Ia endoleak and sac expansion from 55 to 63 mm in diameter that was caused by proximal neck dilation and stent graft migration (Fig. 1a, b). We planned proximal additional stentgraft with bilateral renal artery snorkel technique.

**Fig. 2:**
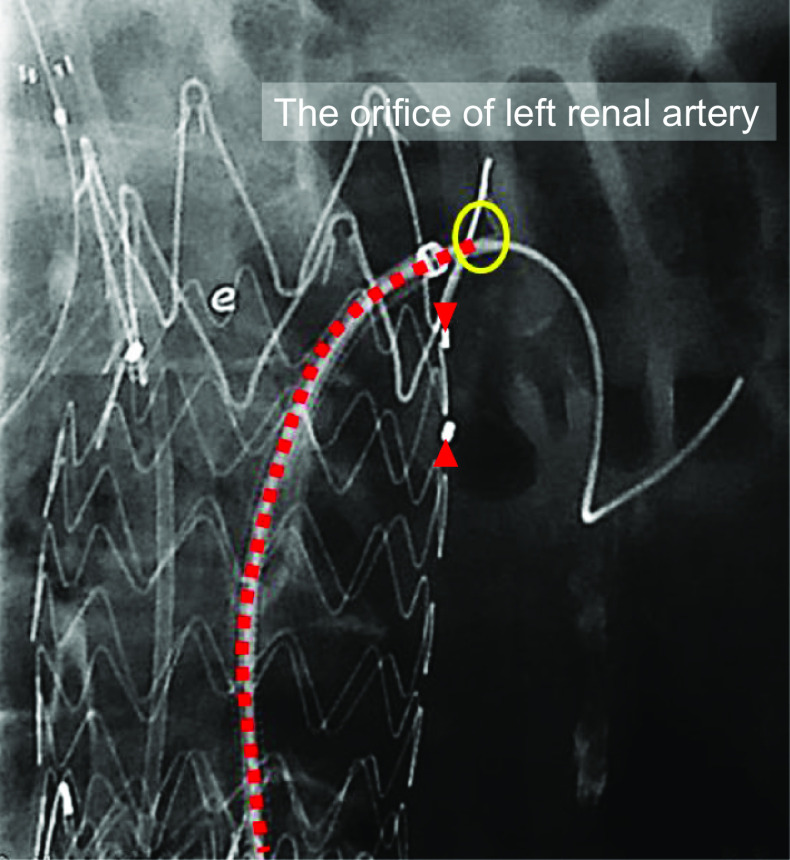
The left renal artery cannulation was done via the trans-femoral access because it was difficult through the trans-brachial access due to interference of the supra-renal stent of the previous Endurant II stentgraft. The ring maker indicates the orifice of the left renal artery and the arrowhead indicates the fenestration to the left renal artery. The ring maker indicates the orifice of left renal artery

**Fig. 3:**
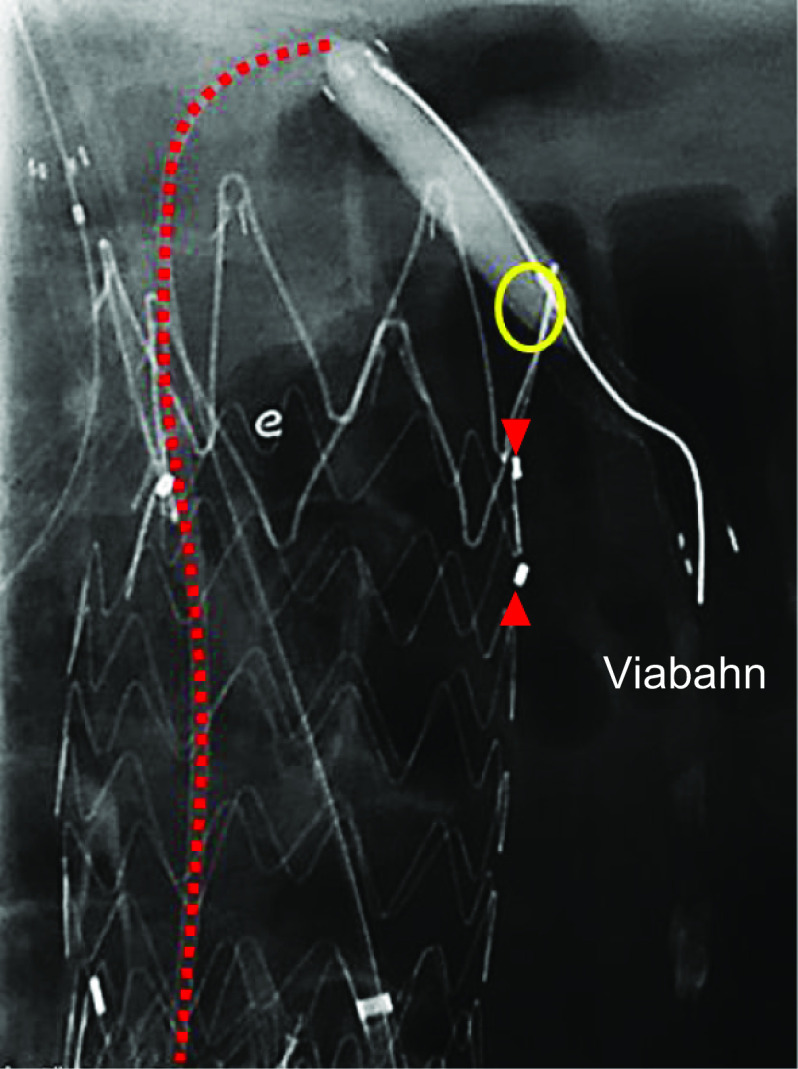
The angioplasty balloon was inflated in the proximal end of the Viabahn endoporosis and pushed up for replacing the proximal end from downward to upward. The ring maker indicates the orifice of the left renal artery and the arrowhead indicates the fenestration to the left renal artery. The ring maker indicates the ﻿orifice of left renal artery

**Fig. 4:**
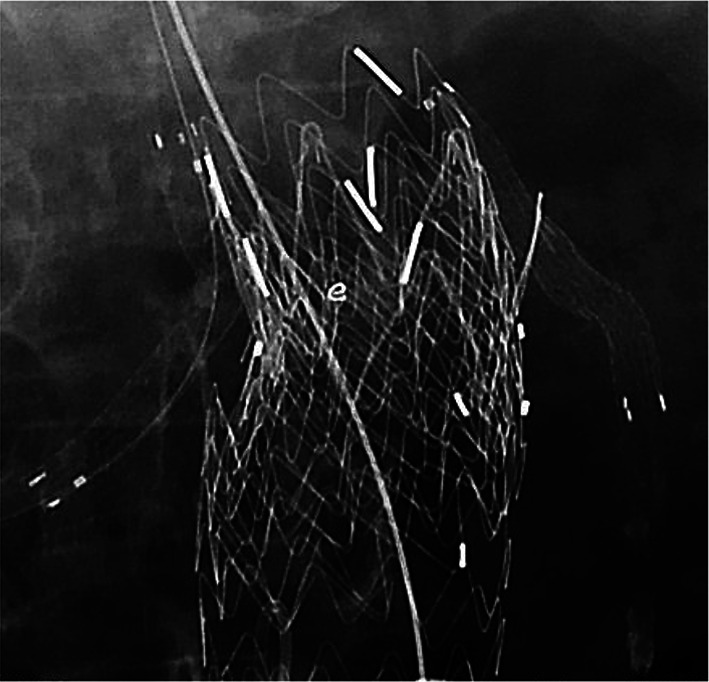
Double snorkel technique was performed with an additional stentgraft cuff deployed parallel to the Viabahn endoporosis

During the secondary intervention however, it was difficult to cannulate to the left renal artery through the trans-brachial access due to interference of the supra-renal stent from the previous Endurant II stentgraft. A 5-mm-diameter Viabahn stentgraft (W.L. Gore, Flagstaff, AZ, USA) was nonetheless able to be delivered into the left renal artery via trans-femoral access with a 5 Fr Destination guiding sheath (Terumo, Tokyo, Japan) (Fig. 2). It was difficult to treat the type Ia endoleak using the periscope technique of the left renal artery because of the previous fenestrated Endurant II stentgraft. Therefore, repositioning the proximal end of the Viabahn was necessary. A 5-mm-diameter SABAR balloon (Cordis, Hialeah, FL, USA) with a 0.018-in. Thruway guidewire (Boston Scientific, Natick, MA, USA) was inserted coaxially through the destination guiding sheath. After balloon touch up was performed over the entire length of the Viabahn, the balloon inflated in the proximal end of the Viabahn and then pushed up to replace the proximal end from down to up (Fig. 3). The additional aortic cuff of a 36-mm-diameter Excluder (W.L. Gore, Flagstaff, AZ, USA) was deployed parallel to the Viabahn (Fig. 4). The final angiography showed no endoleak in the patient’s left renal artery. One year after the additional treatment, CT revealed aneurysm sac shrinkage.

## Discussion

The snorkel technique is an important treatment option for JAAA patients who are unfit for open repair or unsuitable for a fenestrated stentgraft. There are still some situations that could challenge the deployment of abdominal branch stentgraft through the trans-brachial access such as occlusion or tortuosity of the access root or post-arch replacement [3–5]. There are also some complications of trans-brachial access, such as neurological events, temporary occlusion of the left internal mammary artery of post-coronary artery bypass grafting. Therefore, the snorkel stentgraft through trans-femoral access could solve these problems.

The lift snorkel technique, using trans-femoral access has been already described by Mario Lachat in Zurich to treat ruptured JAAA [6]. We report the lift snorkel technique for a type Ia endoleak after FEVAR. They pushed 8Fr sheath over the stiff guidewire, lifting the sheath and snorkel stentgraft upward. However, we used 5Fr sheath without stiff guidewire and pushed only balloon catheter. The technique takes advantage of smaller diameter sheath, and abridgment of a stiff guidewire.

The important point of this technique is the relation between the aortic diameter at the level of the renal artery and intra-aortic length of the snorkel stentgraft. We recommend precise planning and positioning of the snorkel stentgraft prior to deployment because it would be difficult to lift the stentgraft when the intra-aortic length is longer than the aortic diameter.

## Conclusion

The lift snorkel technique is a unique method converting the retrograde approach to antegrade renal artery stenting and would be an effective option for difficult trans-brachial cases for a type Ia endoleak after FEVAR of a JAAA.

## Data Availability

The datasets supporting the conclusions of this article are included within the article.

## Abbreviations

JAAA: Juxtarenal abdominal aortic aneurysm
FEVAR: Fenestrated endovascular aneurysm repair
CT: Computed tomography
